# Metaproteomics reveals functional partitioning and vegetational variation among permafrost-affected Arctic soil bacterial communities

**DOI:** 10.1128/msystems.01238-22

**Published:** 2023-06-05

**Authors:** Samuel E. Miller, Albert S. Colman, Jacob R. Waldbauer

**Affiliations:** 1 Department of the Geophysical Sciences, University of Chicago, Chicago, Illinois, USA; Princeton University, Princeton, New Jersey, USA

**Keywords:** soil, arctic, metaproteomics, permafrost, metabolism, microbial ecology, greening, warming

## Abstract

Microbial activity in Arctic soils controls the cycling of significant stores of organic carbon and nutrients. We studied *in situ* processes in Alaskan soils using original metaproteomic methods in order to relate important heterotrophic functions to microbial taxa and to understand the microbial response to Arctic greening. Major bacterial groups show strong metabolic specialization in organic topsoils. α-/β-/γ-Proteobacteria specialized in the acquisition of small, soluble compounds, whereas Acidobacteria, Actinobacteria, and other detritosphere groups specialized in the degradation of plant-derived polymers. α-/β-/γ-Proteobacteria dominated the expression of transporters for common root exudates and limiting nitrogenous compounds, supporting an ecological model of dependence upon plants for carbon and competition with plants for nitrogen. Detritosphere groups specialized in distinct substrates, with Acidobacteria producing the most enzymes for hemicellulose depolymerization. Acidobacteria was the most active group across the three plant ecotypes sampled—the largely nonvascular, lower biomass intertussock and the largely vascular, higher biomass tussock and shrub. Functional partitioning among bacterial groups was stable between plant ecotypes, but certain functions associated with α-/β-/γ-Proteobacteria were more strongly expressed in higher biomass ecotypes. We show that refined metaproteomic approaches can elucidate soil microbial ecology as well as biogeochemical trajectories of major carbon stocks.

**IMPORTANCE:**

The Arctic is warming twice as fast as the rest of the planet, and Arctic soils currently store twice as much carbon as the entire atmosphere—two facts that make understanding how Arctic soil microbial communities are responding to climate change particularly urgent. Greening of vegetation cover across the Arctic landscape is one of the most prominent climate-driven shifts in Arctic terrestrial ecology, with potentially profound effects on biogeochemical cycling by the soil microbiome. Here we use metaproteomics to document microbial metabolic functions that drive soil carbon and nutrient cycling processes in an Arctic tundra landscape. We identify functional roles among bacterial taxonomic groups that are largely stable across vegetation types, with certain functions strongly expressed by rhizosphere groups reflecting a community metabolic response to greening.

## INTRODUCTION

Arctic soils contain large quantities of organic carbon susceptible to respiration in a warming climate. Though Arctic soils comprise only 16% of soil surface area (1), they hold more than one-third of the global stock of soil organic carbon and roughly twice as much carbon as the entire atmosphere (2, 3). Low temperatures, high soil moisture, and high acidity currently limit the microbial decomposition of abundant, labile soil organic matter (SOM) (4, 5), but with the current trajectory of warming, Arctic soils may release the equivalent of multiple years of anthropogenic C emissions by 2100 (6, 7). Currently, no consensus exists as to whether climate warming will result in net loss of stored soil carbon. Models and meta-analyses indicate either declines (8 - 12) or no net change (13, 14) in soil C stocks, with wide uncertainties (15, 16) especially in the Arctic (17). Arctic warming has widespread ecosystem-level consequences, which affect both the soil and the above-ground plant community in complex, interacting ways (18, 19). For Arctic tundra landscapes, these ecosystem consequences include lengthened growing seasons and increased plant biomass with concomitant changes in rooting depth, plant-microbial interactions, and nutrient demands (14, 20, 21).

Greening of vegetation cover across the Arctic landscape—notably the shift from tussock tundra to taller, higher-biomass woody shrubs—is one of the most prominent climate-driven shifts in Arctic terrestrial ecology, and its consequences for the soil microbial community remain unclear (22 - 24). Long-term greenhouse warming experiments at Toolik Field Station, Arctic Alaska, USA, have documented progressive reduction in topsoil trophic complexity and increases in plant stature, rooting depth, leaf litter accumulation, the evenness of topsoil microbial diversity, and subsoil microbial respiration (25, 26). Greening itself might substantially offset soil C losses (27), since woody plant biomass can store more carbon for a given amount of nitrogen, generally the limiting nutrient during the Arctic growing season (28). Interactions between microbes in the seasonally thawed active layer of the soil and the overlying, progressively greening vegetation are biogeochemically critical, since roughly one-third of Arctic soil carbon is within 1 m of the land surface (2).

Uncertainty in predictions of changes in Arctic soil biogeochemistry is due in part to an incomplete understanding of interactions between microbes, plants, and the physical environment (29 - 31). The structural complexity and occlusion of SOM precludes the *in situ* measurement of most reaction intermediates linking plant inputs to efflux gases, such as CO_2_, via microbial metabolisms (32). One approach to analyzing SOM transformations is the identification of characteristic relationships between microbial taxonomic groups and processes they control (33). Surveys of 16S rRNA diversity in soils have revealed that the relative abundances of high-level bacterial clades strongly correlate with edaphic properties such as acidity and plant productivity (33 - 37). Many genes encoding key heterotrophic processes are distributed among phylogenetically diverse genomes, often varying in their occurrence between closely related strains (38 - 40). Taxa sharing the potential to express a metabolic pathway but exhibiting different growth rates, resuscitation rates from dormancy, or substrate use efficiencies can respond divergently to a given condition, such as substrate concentration (41, 42). The increasing use of methods that more directly interrogate phenotype has begun to clarify functional partitioning among taxa (43). Exometabolomic experiments have shown that rhizosphere bacterial isolates grown on natural mixtures of exudate metabolites preferentially assimilate classes of compounds exuded at different stages of plant development (43, 44). Population-resolved metatranscriptomics has revealed the emergence and succession of carbohydrate-degrading taxa associated with plant growth and detritus addition (45). Stable isotope probing has identified patterns of substrate utilization among both broader and narrower taxonomic groups (46), including transference of the isotopic label between groups over the course of days, suggesting interactions across trophic levels (47).

Some of the aforementioned approaches have the drawback of extrapolating experimental manipulations to *in situ* activity in soil. Metaproteomic analyses can characterize *in situ* protein expression of microbial communities and thereby functional partitioning realized in the environment, with recent studies linking proteins to metagenome-assembled genomes from deeply sequenced soils (48 - 50). The application of metaproteomics to complex ecosystems has been hindered by low identification rates of peptide mass spectra and the challenge of relating peptides to annotated proteins (51, 52). We developed a novel computational pipeline called ProteinExpress to increase the number and quality of protein identifications from complex samples and applied ProteinExpress to Arctic soil samples from Toolik Field Station. We hypothesized that greening will restructure microbial community protein expression patterns, promoting metabolic activities involved in interactions with plant roots and altering soil nutrient cycling. To understand changes in microbial activity associated with greening, soils were analyzed from three plant ecotypes: the lower biomass, largely nonvascular intertussock ecotype and the higher biomass, and largely vascular tussock and woody shrub ecotypes. These metaproteomic data reveal functional traits of major bacterial groups and how they change with vegetation.

## MATERIALS AND METHODS

### Sample collection and proteomic mass spectrometry

Cores of permafrost-affected soil were collected at the end of the lower Arctic growing season (10–12 August 2014) in the vicinity of Toolik Field Station, Alaska, USA (TFS) (Fig. S1; Table S1). Cores were taken from three of the common plant ecotypes of Arctic North American moist acidic tundra: tussock, intertussock, and shrub (53, 54). The sedge, *Eriophorum vaginatum*, forms hemispherical tussocks ~0.5 m in radius, with a dense structure of slender roots extending through the organic soil to the base of the active layer (55, 56). Tussock sedges are spaced ~0.5 m apart and surrounded by diverse, low-lying intertussock vegetation, including mosses, lichens, and prostrate herbaceous plants (57). Woody shrubs ~1 m in height (*Betula nana* and *Salix pulchra*) are abundant around near-surface runoff channels, with nonvascular and herbaceous plants growing in the understory. Shrubs have a higher density of fine roots distributed at shallower depths in the organic layer than tussocks (55, 58). Sampling sites were located in the north–south facing hillslopes just south of TFS and the west facing hillslope of Imnavait Creek valley 12 km east of TFS. The TFS and Imnavait soils (ruptic-histic aquiturbels) developed from glacial outwash deposits of ~55 ka and ~125 ka and have average soil pH values of 4.34 and 4.80, respectively (59, 60). Average TFS soil temperatures at 5 cm and 50 cm depth over the sampling period were 12°C and 5°C, respectively (61). Cores to the permafrost or water table were extracted using a serrated push-corer (6.35 cm inner diameter), returned to TFS on blue ice, and frozen at −80°C within 3 h of sampling. Cores were transported from TFS to the University of Chicago on dry ice and stored at −80°C.

Metaproteomic datasets were collected from 15 organic topsoil samples ~5 cm below the surface and from three mineral subsoil samples ~5 cm below the organic-mineral boundary. Core sectioning, protein extraction, protein digestion, peptide recovery, and LC-MS analysis are detailed in Supplemental Text (doi.org/10.6084/m9.figshare.c.6485806). Protein extraction was based on the method of Chourey et al. (62). Protein digestion and peptide recovery followed a modified eFASP (enhanced Filter-Aided Sample Preparation) procedure (63). LC-MS analysis was performed as in Miller et al. (64). Proteomic mass spectral data is archived in the MassIVE repository under accession MSV000084386.

### Data analysis

#### Overview of ProteinExpress software

We developed a Python 3 application, ProteinExpress (Fig. S7), for protein identification and annotation. ProteinExpress is designed to address the problem of database incompleteness in metaproteomic peptide-spectrum matching and protein inference, which can be a significant issue when the search database consists of population bins of contigs from a paired metagenomic dataset. Genomic microdiversity prevents complete sampling of every sequence variant, even in cold Arctic soils with relatively low diversity compared to warmer soils (65). Additionally, many reads are not assigned to contigs, let alone bins, especially in metagenomes from complex environments (66). Therefore, a match between a peptide mass spectrum and a protein-coding sequence does not guarantee that the mass spectrum originated from the taxon associated with the protein, as opposed to another taxon producing the same peptide in an unsampled coding sequence.

ProteinExpress addresses database incompleteness by first searching mass spectra against both reads and contigs from metagenomes/metatranscriptomes and then aligning matching sequences to the full set of bins representing the major taxonomic groups in the environment. The alignment scores to each bin from sequences assigned the same function (e.g., all sequences with spectral matches annotated as ATP synthase) are used to measure the relative expression levels of the function by the suite of taxa. These scores and the overall relative abundances of proteins, as determined from spectral counts, factor into the metric, Φ, introduced below, which is used to compare the protein expression profiles of taxonomic groups and how a single taxonomic group’s expression profile differs between samples. ProteinExpress uses both new algorithms and existing bioinformatic tools, which are implemented by the software in a pipeline (Fig. S1 and S2). ProteinExpress is publicly available at https://github.com/semiller10/protein-express.

#### Construction of search databases

Peptide sequences were assigned to mass spectra by searching a sequence database generated from publicly available Alaskan soil metagenomic and metatranscriptomic datasets (Table S2), combining sequence data from multiple sites and sequencing projects to achieve representation of the protein sequence space of the sampled Toolik-area soils. We used 12 metagenomes and 6 metatranscriptomes from Imnavait, the area of one of our metaproteomic sampling sites (67), and 10 metagenomes from the CiPEHR site 200 km south of TFS (65). The ProteinExpress pipeline trimmed reads from these 28 2×150 bp datasets with SolexaQA++ dynamictrim at a 1% nucleotide error probability cutoff (68). Full and partial prokaryotic genes were called and translated with Prodigal (69). Predicted proteins from each dataset were substring dereplicated with CD-Hit (70), forming 28 search databases of unassembled sequences from each reference dataset. Contigs were assembled by MEGAHIT from each dataset (minimum *k*-mer length 27, maximum length 87, and step size 10) (71). Using the Graph2Pro pipeline (66), another 28 databases of predicted proteins were produced from the assembled datasets.

We binned contigs from the 12 Imnavait metagenomes sharing a common taxonomic rank, e.g., bins of *Pseudomonas* and Rhizobiales (Table S3; Fig. S7). The method used to construct the bins was designed to maximize both the scope and quality of taxonomic bins by starting with single assemblies of the 12 Toolik-area metagenomes and then rebinning co-assembled reads mapping to the set of preliminary bins with the same taxonomy. Initially, assemblies from each dataset were kept separate. Contigs ≥2,500 bp were binned by MetaBAT 2 on the basis of read coverage, as determined by Bowtie 2 (72), and tetranucleotide frequency (73). Binned contigs were aligned to RefSeq (release 83) using DIAMOND (74), retaining 500 hits per query. The lowest common ancestor was assigned from subject sequences with alignment bit scores within 10% of the top bit score and its taxonomic hierarchy retrieved from the NCBI Taxonomy Database (75). Taxonomic ranks of binned contigs were compared in anvi’o (76). We excluded bins representing taxa, such as Verrucomicrobia, that did not have at least 90% CheckM single-copy gene completeness. The final bins captured ~80% of the prokaryotic diversity in the active layer found in Toolik-area 16S rRNA libraries (Fig. S2). Taxonomically consistent contigs were exported and pooled with contigs sharing the same taxonomic classification from the other datasets. Reads mapping to these contigs were coassembled with MEGAHIT as before, generating a final set of contigs for each taxonomic bin.

#### Proteomic data analysis

The ProteinExpress pipeline used MS-GF+ to search each metaproteomic dataset against the 28 databases of unassembled reads and 28 databases of assembled contigs (Table S1) (77). Each spectrum could return a peptide-spectrum match (PSM) from multiple reads and/or contigs. Database search against assembled nucleotide datasets was refined with the Graph2Pro pipeline (see Supplemental Text at doi.org/10.6084/m9.figshare.c.6485806) (66). PSMs meeting the 1% false discovery rate cutoff from each target-decoy search were selected (78). The one or more full- or partial-length proteins containing a PSM are henceforth called PSM-protein candidates.

PSM-protein candidates were aligned by BLASTp against RefSeq (release 83) to find homologous proteins (79, 80). Of the 500 subject sequences returned per query, those within the top 10% by bit score were retained. If more than five subject sequences were selected, then five were evenly subsampled in descending order of bit score to make the following steps computationally tractable. In the case of a PSM-protein candidate with 500 hits to identical or near-identical RefSeq sequences, hits #1, 125, 250, 375, and 500 would be retained. Selected subject sequences were sorted by NCBI Taxonomy ID into fasta files for Bacteria and Archaea and searched by eggNOG-mapper (via DIAMOND) against superkingdom-level databases of protein Orthologous Groups (see Supplemental Text at doi.org/10.6084/m9.figshare.c.6485806) (81, 82). At this point in the ProteinExpress pipeline, each PSM is associated with a set of PSM-protein candidates, which in turn are associated with up to five functionally annotated RefSeq hits. Only PSMs with identical eggNOG functional description strings (e.g., “Citrate synthase”) among all of the PSM-protein candidate hits were retained. A set of PSMs sharing a functional annotation is called a metaprotein (83).

For purposes of interpretation, we defined 141 Functional Groups of significance for cellular biology and biogeochemical cycles from 2,678 unique pairs of eggNOG gene family and description annotations (Table S4 at doi.org/10.6084/m9.figshare.c.6485806). For example, “CITA” + “Citrate synthase” was assigned to the “TCA Cycle” Functional Group. Some Functional Groups, such as “TCA Cycle,” are equivalent to biochemical pathways. Others, such as “Ribosome,” “Transposase,” and “Ribose Transport,” are comprised of functions not necessarily related through a common pathway. When needed, eggNOG-mapper gene family and functional descriptions were cross-referenced to databases (UniProtKB, InterPro, KEGG Orthology, and CAZy) to assign Functional Groups. For example, sequences with a description of “Hydrolase, family 38” lacking an eggNOG gene family assignment were assigned to “Mannose Cleavage,” as CAZy records that enzymes assigned to Family 38 on the basis of sequence homology are only known to be active on α-linked mannose residues (84).

The relative expression levels of peptides, metaproteins, and Functional Groups were estimated by NSAF (normalized spectral abundance factor) (85):


(1) $${NSAF}_{N}=\frac{D_{N}/L_{N}}{\sum_{i=1}^{n}{(D}_{i}/L_{i})}$$

where *N* is the peptide index; *D_N_* is the number of spectra matched to peptide *N*; *L*_*N*_ is the average length of protein subject sequences returned from BLASTp searches of PSM-protein candidates containing peptide *N* (see above); and *n* is the total number of peptides identified in the dataset. Spectrum count is normalized to protein length, as longer proteins generate more tryptic peptides and are thereby more likely to be observed. The average standard deviation of relative subject sequence lengths from sets of PSM-protein candidates was 2.0%. Metaprotein and Functional Group NSAF values were calculated by summing the NSAF values of peptides associated with the given metaprotein or Functional Group.

We developed a metric, Φ, to estimate relative metaprotein expression levels within and between taxa. Φ takes into account both the relatedness of a metaprotein to a particular bin of contigs (serving as a measure of the likeliness of expression by organisms in the bin) and its NSAF, which represents the level of metaprotein expression irrespective of the bin. To determine Φ, each peptide’s PSM-protein candidates were aligned to each bin by BLASTp. The single candidate that produced the alignment with the lowest E-value was selected, only considering alignments with E-values <0.01. Selection of this candidate ensures the strongest possible nonrandom association between a peptide and each taxonomic bin. The strength of each association was measured by the alignment’s bit score, *S’. S’* was normalized to the range, [0, 1], by subtracting the minimum *S’* among the bins (*S’*_min_) and dividing by *S’*_max_–*S’*_min_. Normalization allows comparison of relative levels of production of a peptide among the taxonomic groups in the sample—which must be well-represented by the bins—with minimum and maximum production by groups corresponding to values of 0 and 1, respectively. The normalized value of *S’* was multiplied by the NSAF value of the peptide in the dataset, producing φ*_N_*, a metric representing both peptide abundance and taxonomic similarity.


(2) $$\left\{ \begin{array}{ll} \varphi_{N}=NSAF_{N}\;\times \;\frac{S^{\prime }-S_{min}^{\prime }}{S_{max}^{\prime }-S_{min}^{\prime }} & if\;E\text{-}value\;of\;peptide\text{-}bin\;alignment<0.01,else\\ \varphi_{N}=0 & \; \end{array}\right.$$

φ*_N_* was extended to metaproteins and Functional Groups (Table S4 at doi.org/10.6084/m9.figshare.c.6485806) by summing φ*_N_* values of PSMs categorized as a certain metaprotein or Functional Group, *P*.


(3) $$\varphi_{P}=\sum_{i}^{n}\varphi_{N,i}$$

We used Φ_bin_, which scales φ*_P_* to the maximum value across bins (φ*_P,_*_max bin_), to compare patterns of functional partitioning between taxonomic groups. We used Φ*_P_*, which scales φ*_P_* to the maximum value across metaproteins or Functional Groups (φ*_P_*_,max function_), to compare a taxonomic group’s protein expression profile between samples. In either case, if the maximum value of φ is 0, Φ is assigned a value of 0. Φ_bin_ and Φ*_P_* lie in the range [0, 1], with at least one value across the set of bins or functions, respectively, equal to 1.


(4) $$\Phi_{bin}=\frac{\varphi_{P}}{\varphi_{P,max\;bin\;}}$$

(5) $$\Phi_{P}=\frac{\varphi_{P}}{\varphi_{P,max\;function}}$$

To understand differences in the functional profiles of taxonomic groups between plant ecotypes, linear discriminant analysis and principal component analysis were conducted with Φ_bin_ and Φ*_P_* values. For analysis of between-ecotype variance of a given taxon’s functional profile, each data point in the multivariate analysis is a taxonomic group with a vector of Φ_bin_ values over all Functional Groups. For analysis of between-ecotype variance of a given function’s distribution between taxa, each data point is a Functional Group with a vector of Φ*_P_* values over all taxonomic groups.

## RESULTS

### Identification of proteins and activity of taxonomic groups

We identified peptide sequences for 3,142–16,235 MS2 spectra (PSMs) from each of the 15 organic and 3 mineral soil samples, of which 1,079–5,003 (27.1–36.9%) were functionally annotated (Fig. S1; Table S1). Metaproteins, or groups of PSM-containing proteins with the same functional annotation, were compared to 12 bacterial taxonomic bins identified from Toolik-area topsoil metagenomes (Tables S2 and S3); prior work indicates that similar sets of taxa are present in the organic and mineral layers of tundra soils (86) and over the course of the growing season (87), albeit at differing relative abundances. The taxonomic specificity of the bins ranged from genus to phylum level. Bins represented the same phylum or class as 81.5% of the reads on average from nine Toolik-area 16S rRNA datasets (Fig. S2; 87 - 91) with Verrucomicrobia, Firmicutes, other lower abundance phyla, and unclassified reads not represented by bins.

Our calculated metric, Φ_bin_ (see Equation 4), was used as a measure of the relative expression of a given metaprotein by different taxonomic groups. We identified overarching patterns in functional partitioning by the 12 major bacterial groups using *k*-means clustering of all Functional Group (Table S4 at doi.org/10.6084/m9.figshare.c.6485806 ) Φ_bin_ values averaged across all organic soil samples, irrespective of plant ecotype. A breakpoint in the reduction of the sum of squared errors with the addition of clusters exists at *k* = 3 clusters (Table S5 at doi.org/10.6084/m9.figshare.c.6485806), which showed consistent expression patterns between taxonomic bins. Protein expression of Functional Groups in Cluster 1 was largely attributed to α-/β-/γ-Proteobacteria; proteins in Cluster 2 were expressed most strongly by Acidobacteria, but also at high levels by α-/β-/γ-Proteobacteria; and functions in Cluster 3 were dominated by Acidobacteria. Other taxa, including Actinobacteria, Bacteroidetes, and Myxococcales (δ-Proteobacteria), produced fewer proteins for most functions.

**Fig 1:**
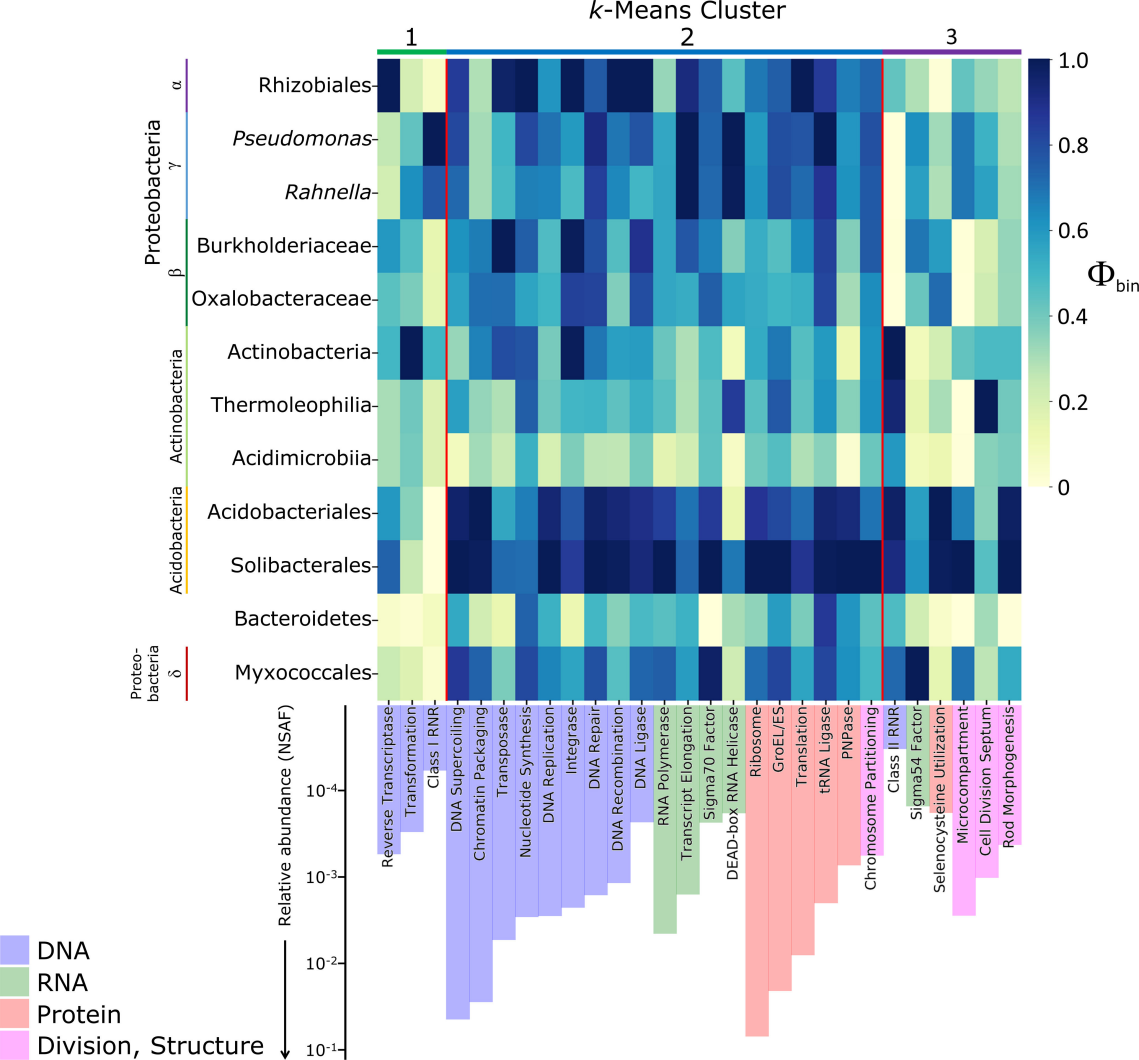
Cell growth-related Functional Group Φ_bin_ values (heatmap) and average overall relative abundances (bars) across organic soil samples. Columns are ordered by cluster assignment, then by Functional Group category (bar color), then by overall relative abundance (NSAF). Φ_bin_ values are column-normalized to between 0 and 1 based on Φ_bin,max_ for that column.

Many core cellular functions, including DNA replication and repair, transcription, translation, and cell division, fall into Cluster 2—being most highly expressed by Acidobacteria and secondarily by α-/β-/γ-Proteobacteria (Fig. 1)—suggesting that these two groups are highly active in the organic soils. Ribosomal proteins and chaperones, along with nucleoid structuring proteins, are among the most abundant proteins in the dataset (NSAF values >0.01; Fig. 1), reflecting the high metabolic and nutrient cost of central housekeeping functions. Cell envelope-related functions (Fig. S3) showed broadly similar distributions to core functions, being most highly expressed by Acidobacteria and α-/β-/γ-Proteobacteria. As expected, gliding motility proteins associated with Bacteroidetes and Myxococcales were found to be expressed essentially solely by those groups, validating the taxonomic specificity of protein assignment by the ProteinExpress pipeline and suggesting that taxon-specific functions were sufficiently represented in our sequence bins.

We also examined the expression of Functional Groups when normalized to that of ribosomal proteins (by subtracting Φ_bin,Ribosome_ values, as a proxy for overall cellular activity; Fig. S4). Taxonomic expression profiles with marked differences in ribosomal proteins suggest that the function was not expressed in proportion to an overall activity of the different taxonomic groups. This comparison reinforces the result from clustering that many functions were skewed strongly either toward (Cluster 1) or away (Cluster 3) from α-/β-/γ-Proteobacteria.

### Carbon cycling and energy conservation

**Fig 2:**
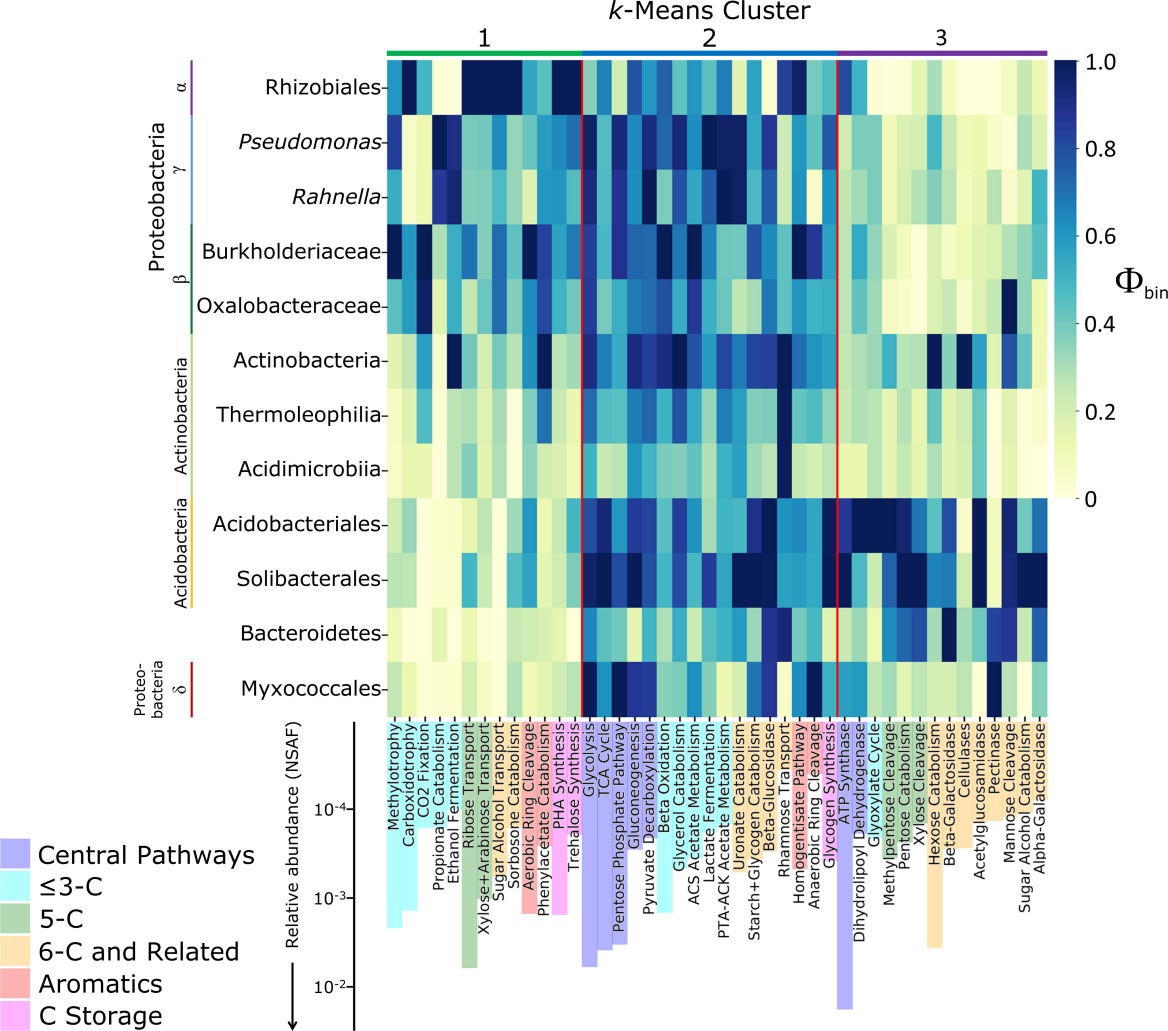
Carbon-related Functional Group Φ_bin_ values (heatmap) and average overall relative abundances (bars), across organic soil samples. Columns are ordered by cluster assignment, then by Functional Group category (bar color), then by overall relative abundance (NSAF). Φ_bin_ values are column-normalized to between 0 and 1 based on Φ_bin,max_ for that column.

Acidobacteria, class Actinobacteria, Bacteroidetes, and Myxococcales dominated the expression of extracellular enzymes responsible for depolymerization of plant cell wall polysaccharides (Fig. 2). Acidobacteria had the highest Φ_bin_ values for most core C metabolism and energy conservation functions, including the TCA cycle and ATP synthase. The group strongly expressed enzymes acting on diverse heteropolysaccharide bonds, including β-1,4-linked D-xylose, β-1,4-linked D-glucose, and β-1,4-linked D-mannose, and likewise expressed enzymes required for the metabolism of diverse pentoses, such as xylose and arabinose. Acidobacteria also produced the highest levels of enzymes involved in the breakdown of starch and glycogen, including α-amylase, 4-α-glucanotransferase, amylo-α-1,6-glucosidase, and α-glucosidase. Overall, these protein expression patterns point toward catabolism of hemicelluloses, starch, and glycogen as the main carbon and energy sources for the highly active Acidobacteria in these soils.

C metabolism protein expression patterns of other bacterial groups indicate specialization for breakdown of other plant-derived substrates. Actinobacteria dominated the expression of endoglucanase and cellobiosidase, enzymes required for the debranching and cleavage of oligosaccharides from cellulose. Bacteroidetes and Myxococcales were distinguished from Acidobacteria and Actinobacteria by strong expression of pectinases. Pathways such as the protocatechuate-4,5 cleavage pathway for the aerobic degradation of heterogeneous aromatic compounds derived from lignin were most heavily expressed by Burkholderiaceae. Anaerobic aromatic degradation pathways had significantly lower levels overall, which was generally true of anaerobic versus aerobic pathways in our samples. Lactate and ethanol fermentation enzymes were about two orders of magnitude less abundant than TCA cycle enzymes and ATP synthase, and terminal electron acceptors besides O_2_ are relatively scarce in organic-rich tundra soils (92).

Different taxonomic groups dominated the expression of pathways required for utilization of small soluble molecules and gases. Carboxidotrophy and methylotrophy had relatively high overall abundances, with Rhizobiales dominating the expression of CO dehydrogenase, and β- and γ-Proteobacteria most strongly expressing methanol dehydrogenase (MxaF) and formaldehyde oxidation pathways. The nondetection of methane monooxygenase, low representation of Archaea in nucleotide datasets, and identification of only three metaproteins with strong alignments to Archaea in RefSeq indicate that biogenic methane was not a significant substrate. Rhizobiales produced the highest levels of monosaccharide transporters but low levels of enzymes for polysaccharide depolymerization, glycolysis, and the catabolism of nonglucose monosaccharides. Rhizobiales dominated the expression of enzymes for polyhydroxyalkanoate synthesis, a C storage mechanism with higher overall abundance than the other identified C storage pathways of trehalose and glycogen synthesis.

### Nutrient cycling

**Fig 3:**
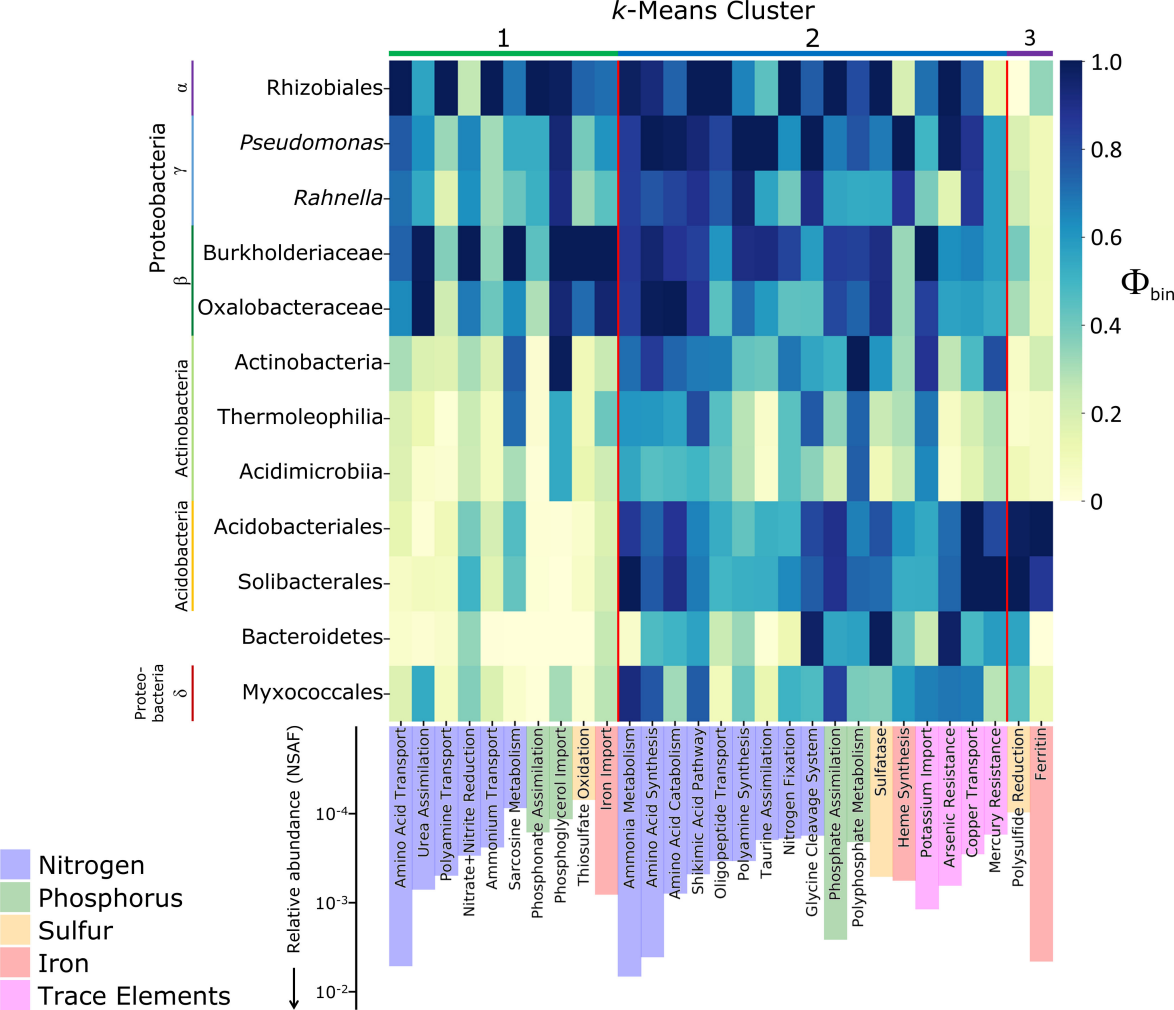
Nutrient-related Functional Group Φ_bin_ values (heatmap) and average overall relative abundances (bars), across organic soil samples. Columns are ordered by cluster assignment, then by Functional Group category (bar color), then by overall relative abundance (NSAF). Φ_bin_ values are column-normalized to between 0 and 1 based on Φ_bin,max_ for that column.

Proteobacteria—particularly Rhizobiales—dominated the production of N transporters (Fig. 3). Proteins involved in amino acid transport had a higher overall abundance than those involved in oligopeptide and polyamine transport and urea assimilation, which in turn were more abundant than proteins involved in ammonium transport, nitrate/nitrite reduction, and N_2_ fixation. Intracellular N cycling functions, such as amino acid synthesis and catabolism pathways, were much more evenly expressed between taxonomic groups. The abundant ammonia metabolism Functional Group, which largely consists of the glutamine synthetase metaprotein, was more strongly expressed by Acidobacteria.

Phosphate acquisition proteins, including phosphate transporters and phosphatases, were higher in overall abundance than polyphosphate and phosphonate acquisition proteins, and were relatively evenly expressed across groups. Iron is known to be an important substrate in microbial iron mats that float on submerged soils around Toolik (93). In our soils, none of which were submerged, Fe-related functions were relatively abundant but not clearly related to dissimilatory Fe metabolism, which was not identified. Ferritins were especially abundant and overwhelmingly produced by Acidobacteria. Heme synthesis pathways were most heavily expressed by γ-Proteobacteria, and proteins involved in Fe import, largely through siderophore synthesis, were most strongly expressed by β-Proteobacteria. These functions had a more even taxonomic distribution than ferritin.

### Differences between plant ecotypes

**Fig 4:**
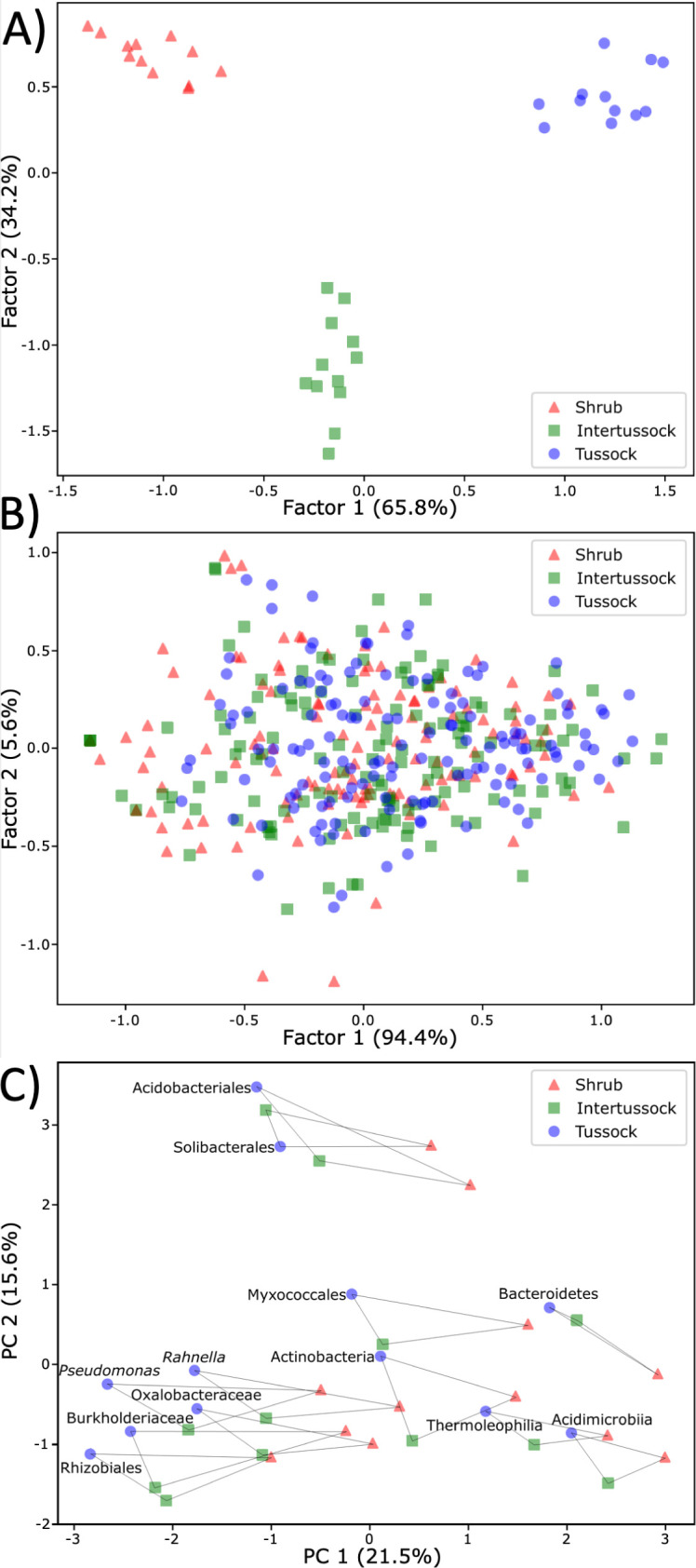
Linear discriminant analyses for organic soil samples between plant ecotypes of (A) Φ_bin_ values, with each data point representing one of 12 taxonomic bins in a given ecotype and (B) Φ_Functional Group_ values, with each data point representing one of 141 Functional Groups in a given ecotype. The percentage is the proportion of separation due to the discriminant function. (**C**) Principal component analyses of Functional Group Φ_bin_ values, showing principal components 1 and 2 (percentage indicates the proportion of variance explained by each PC), with lines connecting points representing the same taxonomic group in different plant ecotypes.

Multivariate analyses of Φ were used to understand differences in the functional profiles of taxonomic groups between plant ecotypes. Complementary linear discriminant analyses of Φ_bin_ and Φ_Functional Group_ values reveal that organic soil samples cluster by plant ecotype when considering the functional profiles of taxonomic groups (Fig. 4A) but not the taxonomic profiles of Functional Groups (Fig. 4B)—taxonomic groups maintained functional specialization across plant ecotypes, but the expression levels of functions varied across ecotypes. This result was supported by principal component analysis of Φ_bin_ values (Fig. 4C), which shows that taxonomic groups generally maintained their relative positions in functional space while the whole community of all taxonomic groups shifted systematically between environments.

Four Functional Groups in tussock and intertussock organic soil samples were found to have statistically significant differences (Welch’s t-test) in overall relative abundance between environments (shrub is ignored due to the fewer number of metaproteomic datasets than tussock and intertussock): ribose transport (*P* = 0.00021), xylose+arabinose transport (*P* = 0.019), sugar alcohol transport (*P* = 0.014), and succinoglycan synthesis (*P* = 0.014) (Fig. S5; Supplemental Text at doi.org/10.6084 /m9 .figshare.c.6485806). These functions were more highly represented in tussock than intertussock samples and were most strongly expressed by Rhizobiales. Linear discriminant analysis of the NSAF data (Supplemental Text at doi.org/10.6084 /m9 .figshare.c.6485806) shows that organic soil samples distinctly cluster by plant ecotype, whereas the three mineral soil samples, one from each plant ecotype, form a separate cluster (Fig. S6).

## DISCUSSION

**Fig 5:**
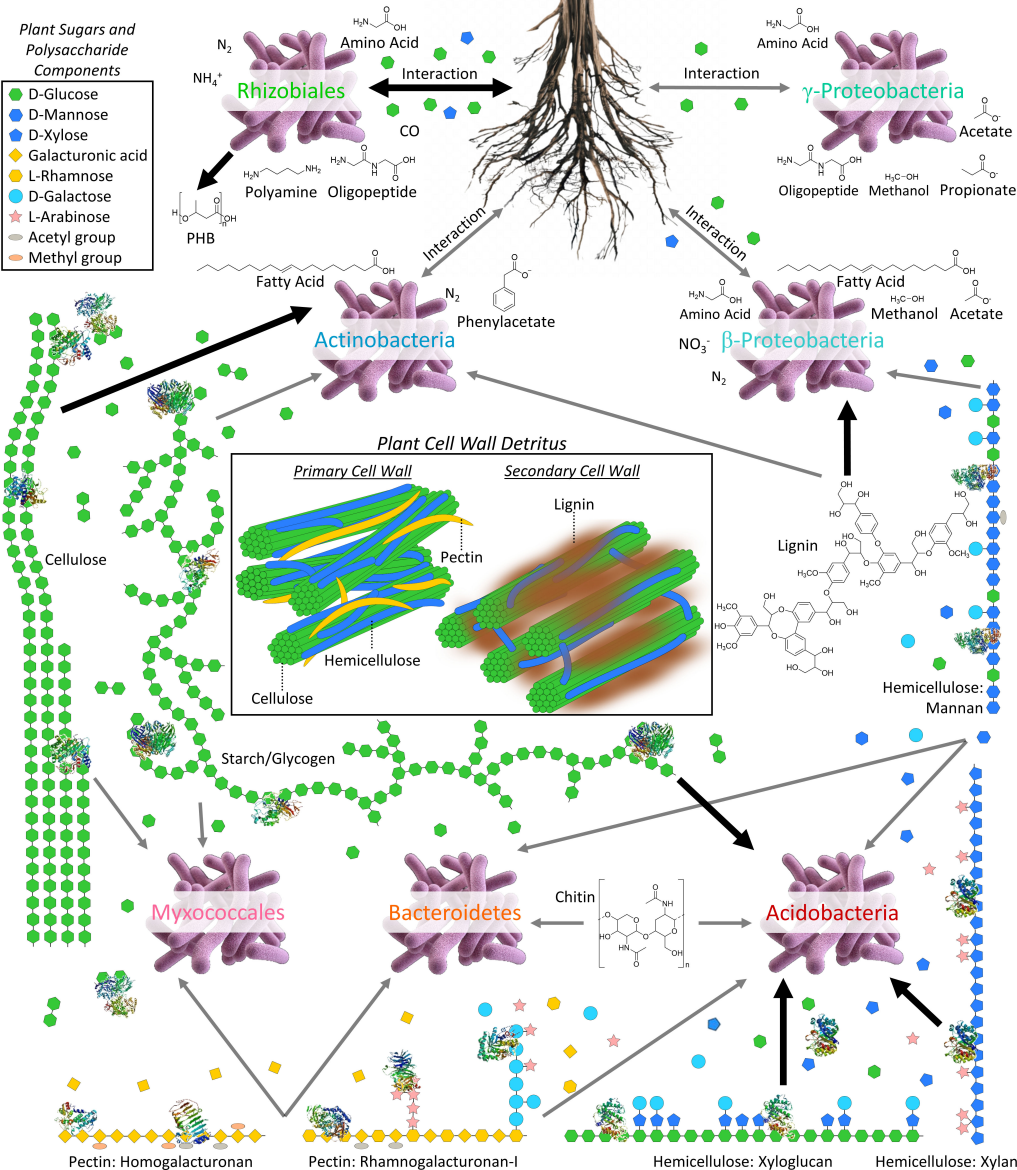
Summary of resource partitioning by major taxonomic groups in organic soils, as determined from Φ_bin_ data.

We profiled microbial protein expression in Arctic Alaskan soils using a metaproteomic approach that accounts for sequence microdiversity, finding that major heterotrophic functions are consistently partitioned among broad taxonomic groups (Fig. 5). While this study was limited to a snapshot view of the late Arctic growing season, these data elucidate functional specialization among microbial taxa, clarifying how each group contributes to the breakdown of plant-derived organic carbon and to soil nutrient cycling. Acidobacteria, the most active group (as indicated by expression of ribosomal proteins, chaperones, and other abundant core proteins; Fig. 1), specialized in the degradation of relatively labile polysaccharides (Fig. 2). In accordance with findings from soils along a permafrost thaw gradient (48) and in *Sphagnum* peat (94), Acidobacteria produced a full complement of enzymes for xylan depolymerization and xylose assimilation: endo-/exo-xylanases, acetyl xylan esterase, xylose isomerase, and xylulokinase. We additionally found that Acidobacteria strongly expressed numerous glycosidases for the breakdown of diverse hemicelluloses and pectins, although Myxococcales and Bacteroidetes dominated the production of pectinases required for depolymerization of pectin’s galacturonan backbone.

In these Toolik-area soils, Actinobacteria dominated cellulase production, in contrast to the Swedish soils in which Acidobacteria predominated, while Actinobacteria had high genomic potential for cellulases but negligible production of transcripts and proteins (48). Our results are consistent with the ability of the majority of Acidobacteria in pure culture to use xylan or starch but not crystalline cellulose or carboxymethylcellulose as a carbon source (95). A metatranscriptomic investigation of Svalbard peat soils also shares certain patterns of functional partitioning with those we observe at Toolik, including the importance of Actinobacteria in the expression of cellulases, Bacteroidetes in branched polysaccharide glycosidases, and Proteobacteria in phenolic degradation pathways (96).

α-/β-/γ-Proteobacteria specialized in the acquisition of small, soluble compounds, including monosaccharides, despite expressing very low levels of glycosidases. These taxa appear to be adapted primarily to the rhizosphere or other soil hot spots receiving pulses of labile carbon substrates or nutrients (97). At the time when we sampled for this study, relatively late in the growing season, labile C compounds from root exudates may have been largely depleted, contributing to the observed lower apparent activity of Proteobacteria compared to Acidobacteria. Rhizobiales (α-Proteobacteria) dominated the expression of transporters for low molecular weight compounds that are among the most abundant types of root exudates (98) as well as proteins for the production of succinoglycan, an exopolysaccharide known to be essential for interactions between nodulating rhizobia and their leguminous hosts (99, 100). Monosaccharide and sugar alcohol transporters and succinoglycan production were the functions that increased most significantly in relative abundance between our soils with low and high root biomass. Furthermore, Rhizobiales dominated the production of CO dehydrogenase for CO oxidation, a process that removes most CO produced by roots, a major source in soils (101, 102). Trace atmospheric CO may also provide a carbon source for subsistence in the absence of root exudates (103), which could be a widespread condition among Rhizobiales in Arctic soils in light of their high expression of the synthesis pathway for polyhydroxyalkanoates, polymers used for C and energy storage and metabolized during later periods of low plant productivity (104).

α-/β-/γ-Proteobacteria appeared to compete strongly with plants for scarce N in Toolik-area soils. By the time we sampled at the end of the growing season, bioavailable N—which is mainly in organic forms—has been found to fall to nearly undetectable levels in soils around Toolik (105). Two mechanisms have been proposed to explain pervasive N limitation of plant productivity in Arctic soils: slow microbial decomposition generating little bioavailable N and high N use efficiency by rhizosphere microbes, with the latter hypothesis supported by ^15^N dilution experiments (106) and meta-analysis of N turnover rates (107). In our datasets, α-/β-/γ-Proteobacteria dominated the production of transporters for nitrogenous compounds, including amino acids, oligopeptides, polyamines, urea, and ammonium, whereas proteins involved in intracellular N cycling—glutamine synthetase being the most abundant—had relatively even expression between Proteobacteria and detritosphere groups such as Acidobacteria (Fig. 3). High N uptake efficiency among α-/β-/γ-Proteobacteria may be driven by depletion of N around growing root tips and demand for nutrients to generate biomass from influxes of root exudates or other soluble C sources (97, 98, 108). Our observation of cells poised for N assimilation agrees with the rapidly responding copiotrophic behavior of Proteobacteria in N fertilization experiments (109). We found that P transporters, in contrast, were more evenly expressed across taxonomic groups, which may be attributed to lower demand relative to available supplies, as Arctic soils are less often P limited.

Bacterial protein relative abundances differed between plant ecotypes in organic soils (Fig. 4A), yet functional partitioning across bacterial groups remained relatively invariant (Fig. 4B)—implying that taxa have consistent metabolic roles in soils supporting different types of vegetation, but the relative prominence of those various roles shifts. A 16S rRNA study of nearby soils documented a shift toward Proteobacteria and away from Acidobacteria in shrub versus tussock soils (87). It was inferred that copiotrophic Proteobacteria were better adapted to use the labile C pool in shrub soils (110). Given that we found higher relative abundances of α-/β-/γ-Proteobacterial functions in highly rooted tussock than sparsely rooted intertussock soils, it may be that greater root density and plant activity generally leads to the formation of rhizosphere hot spots favorable for Proteobacteria. Interestingly, the protein expression profiles of bacterial groups shifted with plant ecotype in the same directions along principal component axes (Fig. 4C), which may signify that different plant stimuli and inputs affected the expression of functions independent of whether those functions were expressed at high or low levels by particular bacterial groups. Lastly, the relatively uniform profiles of overall functional abundance in deeper mineral soils (Fig. S6) may be explained by higher representation of core cellular functions versus more specialized metabolism in relatively inactive cells or the advective homogenization of plant inputs from nearby ecotypes (111).

Patterns of functional partitioning that we found among major bacterial groups seem likely to persist as plant ecotypes continue to shift in response to Arctic warming. These patterns may also apply over large areas of the Arctic, as suggested by the occurrence of nearly identical metagenome-assembled genomes hundreds of kilometers apart (65). Building from the snapshot of the late Arctic summer season presented here, assaying potential shifts in functional partitioning over the full course of the growing season (and during wintertime, increasingly recognized as crucial to Arctic soil C cycling (112)) will test the broader applicability of these observations. Our study particularly highlights the metabolic interplay between rhizosphere α-/β-/γ-Proteobacteria and detritosphere groups such as Acidobacteria in determining the fate and rate of transformation of plant-derived organic carbon in Arctic soils. Understanding the biogeography of functional roles of the full microbial community across seasons and in diverse soils will be crucial to robust predictions of the fate of Arctic soil carbon stocks.

## SUPPLEMENTAL MATERIAL

Fig. S1 msystems.01238-22-s0001.pdf Map of sampling locations in the vicinity of Toolik Lake, Alaska. See Table S1 for details of individual sample numbers.

Fig. S2 msystems.01238-22-s0002.pdf 16S rRNA datasets from organic soils in the area of Toolik Field Station, collected during the growing season from June–August (55-59). Taxa above the red line in the legend are represented by metagenomic bins, whereas those below the line, as well as any other low abundance taxa not listed, are not represented by bins.

Fig. S3 msystems.01238-22-s0003.pdf Cell envelope-related Functional Group Φ_bin_ values (heatmap) and average overall relative abundances (bars) across organic soil samples. Columns are ordered by cluster assignment, then by Functional Group category (bar color), and then by overall relative abundance (NSAF). Φ_bin_ values are column-normalized to between 0 and 1 based on Φ_bin,max_ for that column.

Fig. S4 msystems.01238-22-s0004.pdf Functional Group Φ_bin_ values with Φ_bin,Ribosome_ values subtracted, and average overall relative abundances (bars) across organic soil samples. Columns are ordered by cluster assignment, then by Functional Group category (bar color), and then by overall relative abundance (NSAF). A) Cell growth-related Functional Groups; B) Carbon-related Functional Groups; C) Nutrient-related Functional Groups; D) Cell envelope-related Functional Groups.

Fig. S5 msystems.01238-22-s0005.pdf Overall expression levels (NSAF) of Functional Groups associated with A) DNA, B) RNA, C) translation, D) central carbon metabolism and energy conservation, E) polysaccharide degradation, F) carbon metabolism, and G) nitrogen, H) sulfur, I) phosphorus, J) trace elements, K) stress, L) membrane and wall synthesis, M) movement, N) cell division and structure, and O) other functions.

Fig. S6 msystems.01238-22-s0006.pdf Linear discriminant analyses of NSAF data from metaproteins defined by multiple functional annotation systems: A) GO terms, B) KEGG Orthology IDs, C) Gene Families + eggNOG descriptions, and D) Functional Groups of Gene Family + eggNOG description metaproteins. Each point is a different sample, with both organic (unlabeled points) and mineral soils (labeled) considered. The percentage is the proportion of separation due to the discriminant function.

Fig. S7 msystems.01238-22-s0007.pdf Flowcharts of metaproteomic data analysis in ProteinExpress and of taxonomic bin construction from metagenomes.

Table S1 msystems.01238-22-s0008.pdf Metaproteomic sample information. For map of sample locations, see Figure S1. “NA” in “Depth to water table (cm)” signifies that the water table was at the base of the active layer.

Table S2 msystems.01238-22-s0009.pdf Metagenomic and metatranscriptomic sample datasets used for metaproteomic search database construction. Asterisks indicate data sets that were used in binning.

Table S3 msystems.01238-22-s0010.pdf Taxonomic bins constructed from metagenomic data, taxonomic ranks, and number of contigs (all ≥2,500 nt) per taxonomic bin.

ASM does not own the copyrights to Supplemental Material that may be linked to, or accessed through, an article. The authors have granted ASM a non-exclusive, world-wide license to publish the Supplemental Material files. Please contact the corresponding author directly for reuse.
