# Supplementary figures and images for: Metaproteomics reveals functional partitioning and vegetational variation among permafrost-affected Arctic soil bacterial communities
Source: mSystems. 2023 Jun 5;8(3):e01238-22. doi: 10.1128/msystems.01238-22 (PMC10308928; doi:10.1128/msystems.01238-22)

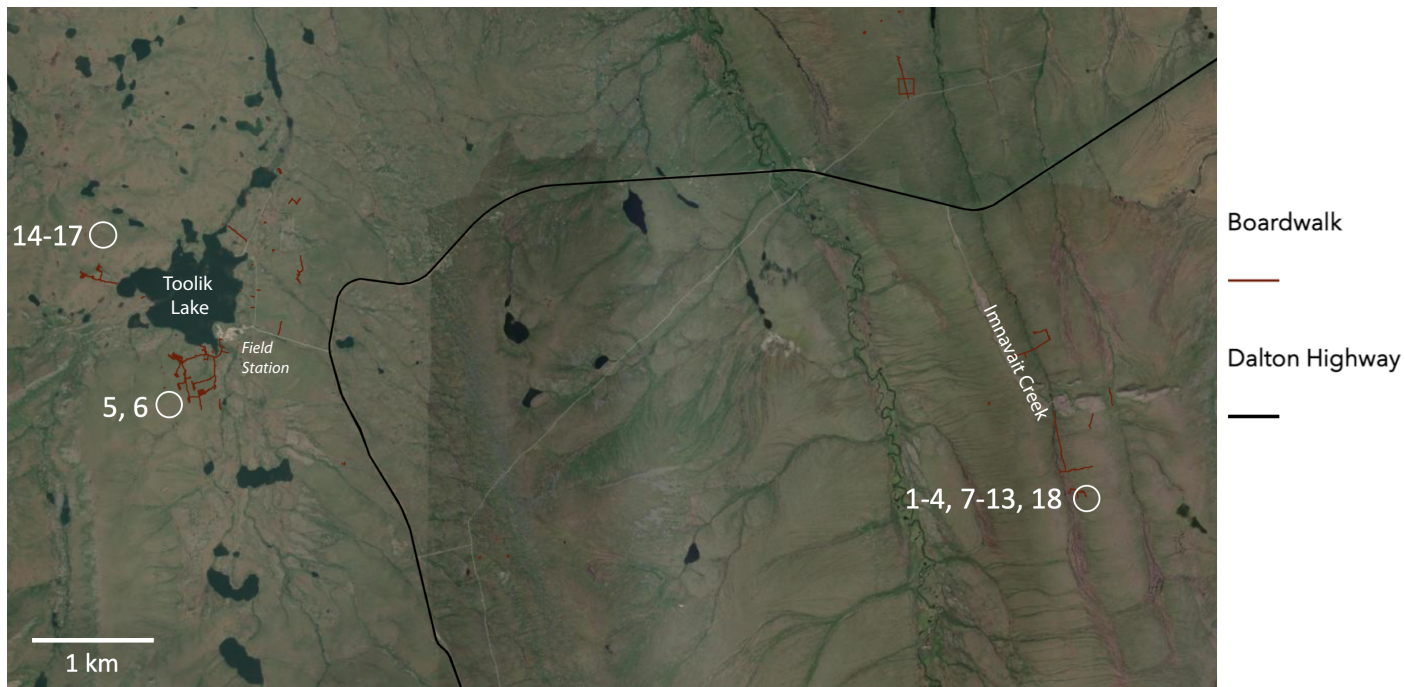

Supplement: Fig. S1 — Map of sampling locations in the vicinity of Toolik Lake, Alaska. See Table S1 for details of individual sample numbers. [file msystems.01238-22-s0001.pdf]

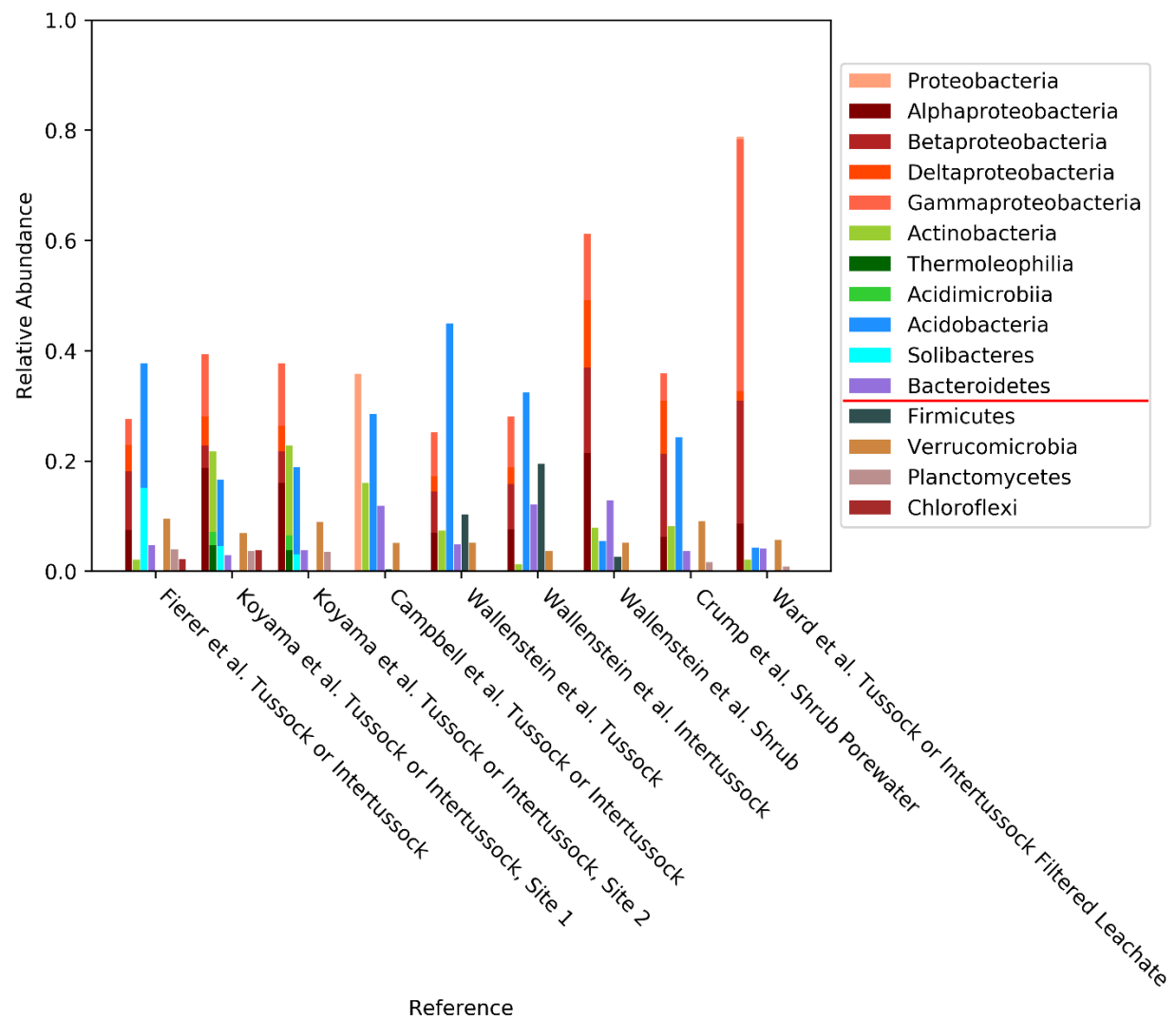

Supplement: Fig. S2 — 16S rRNA datasets from organic soils in the area of Toolik Field Station, collected during the growing season from June–August (55-59). Taxa above the red line in the legend are represented by metagenomic bins, whereas those below the line, as well as any other low abundance taxa not listed, are not represented by bins. [file msystems.01238-22-s0002.pdf]

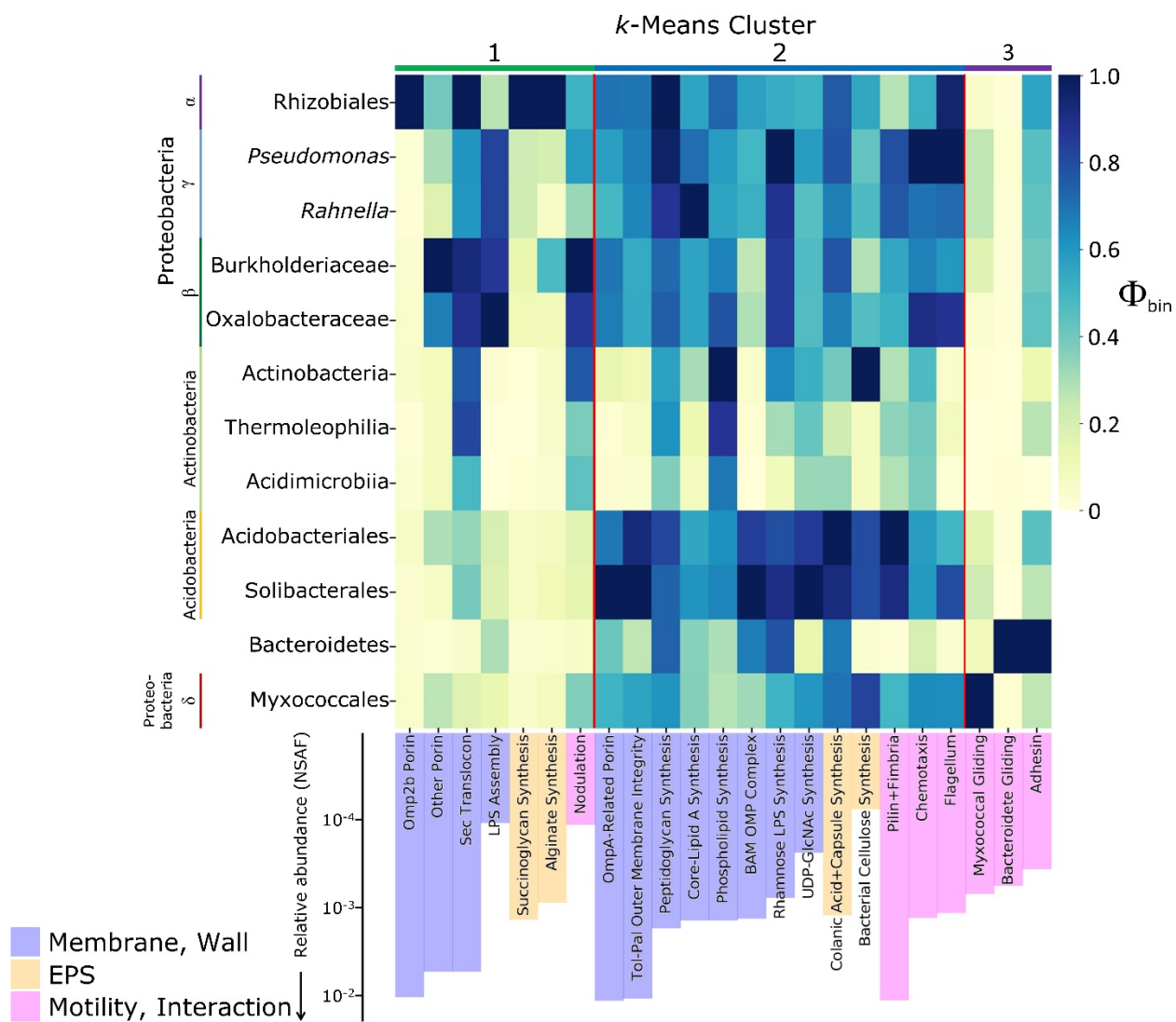

Supplement: Fig. S3 — Cell envelope-related Functional Group Φbin values (heatmap) and average overall relative abundances (bars) across organic soil samples. Columns are ordered by cluster assignment, then by Functional Group category (bar color), and then by overall relative abundance (NSAF). Φbin values are column-normalized to between 0 and 1 based on Φbin,max for that column. [file msystems.01238-22-s0003.pdf]

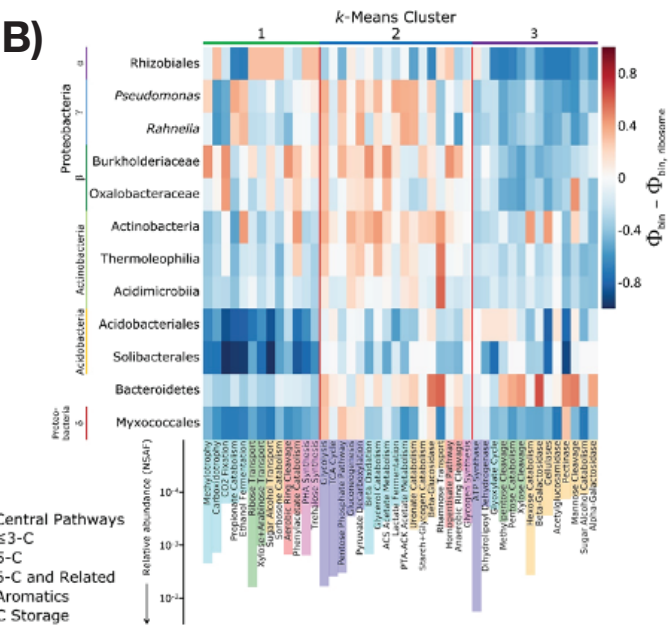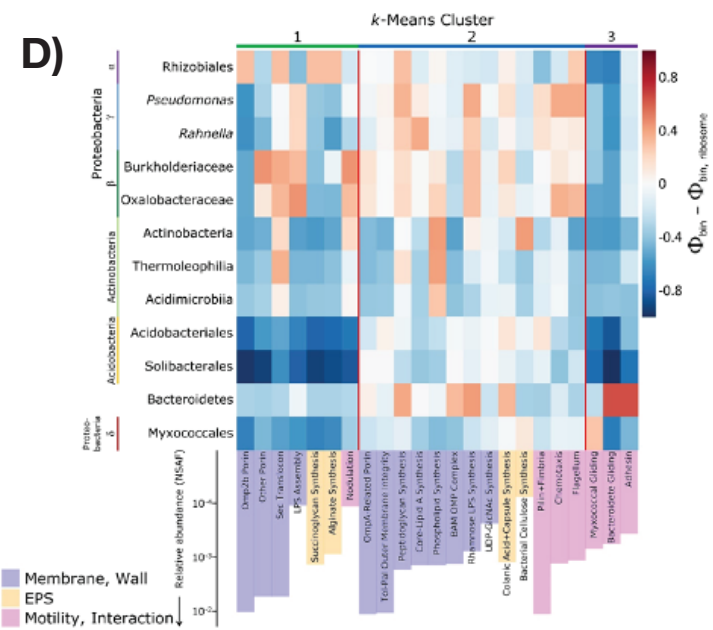

Supplement: Fig. S4 — Functional Group Φbin values with Φbin,Ribosome values subtracted, and average overall relative abundances (bars) across organic soil samples. Columns are ordered by cluster assignment, then by Functional Group category (bar color), and then by overall relative abundance (NSAF). A) Cell growth-related Functional Groups; B) Carbon-related Functional Groups; C) Nutrient-related Functional Groups; D) Cell envelope-related Functional Groups. [file msystems.01238-22-s0004.pdf]

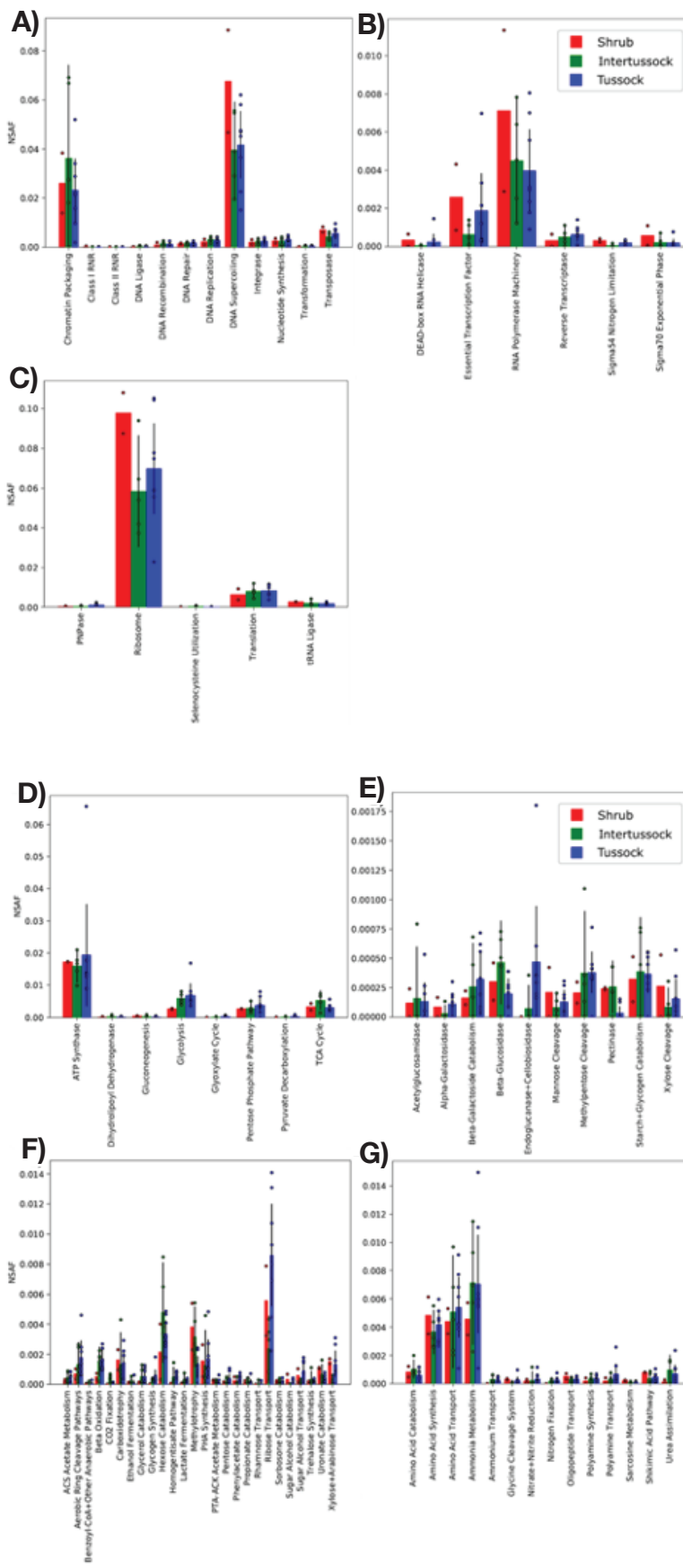

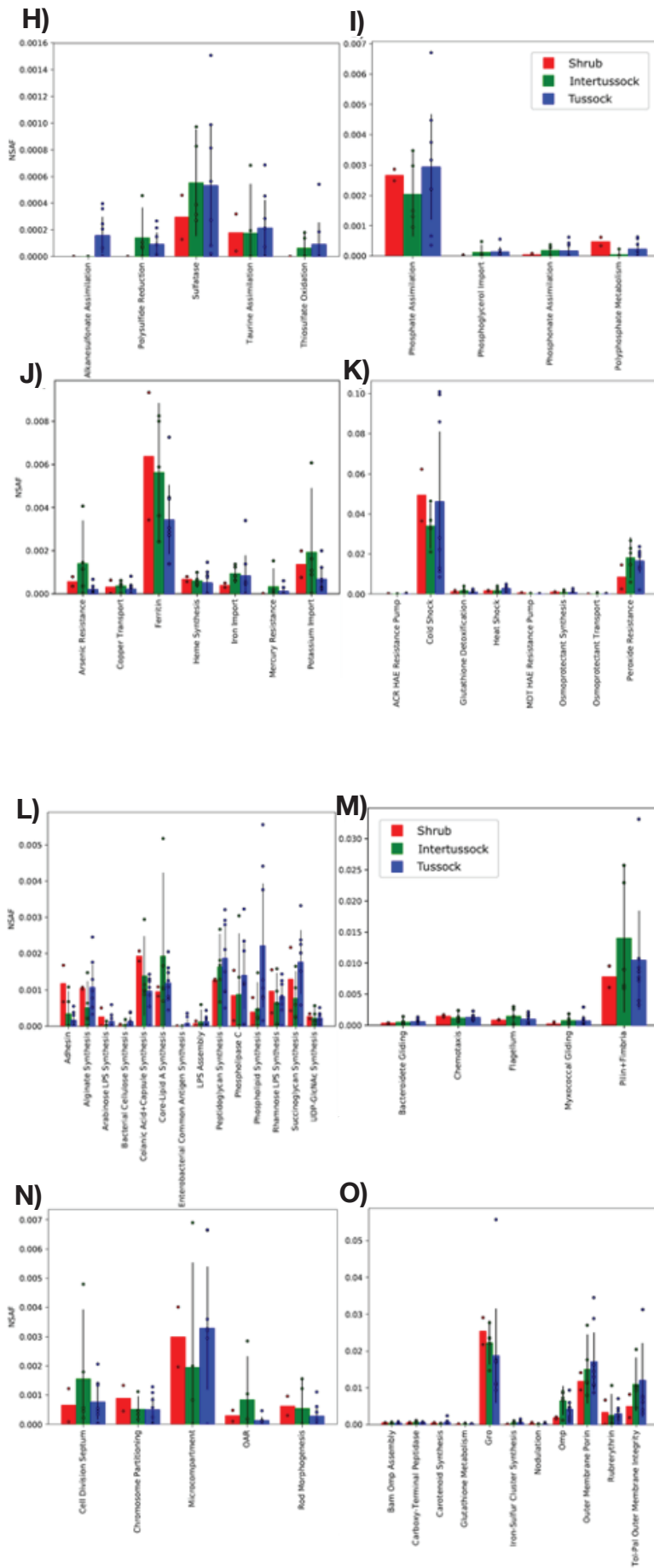

Supplement: Fig. S5 — Overall expression levels (NSAF) of Functional Groups associated with A) DNA, B) RNA, C) translation, D) central carbon metabolism and energy conservation, E) polysaccharide degradation, F) carbon metabolism, and G) nitrogen, H) sulfur, I) phosphorus, J) trace elements, K) stress, L) membrane and wall synthesis, M) movement, N) cell division and structure, and O) other functions. [file msystems.01238-22-s0005.pdf]

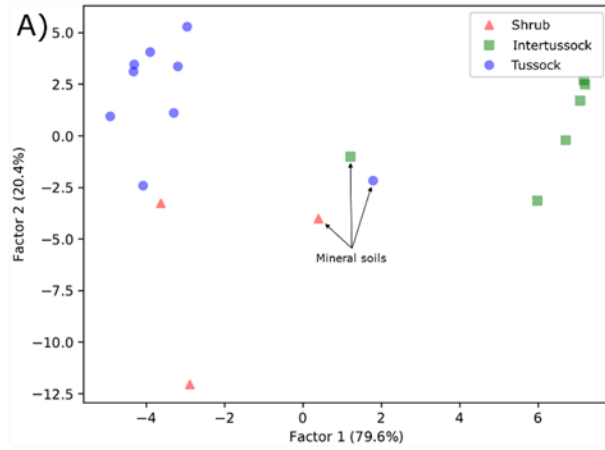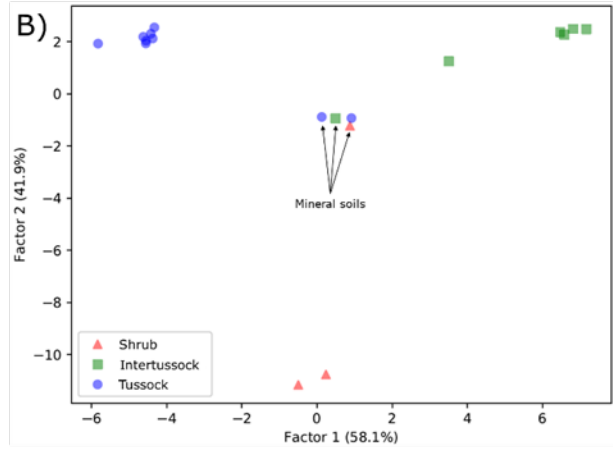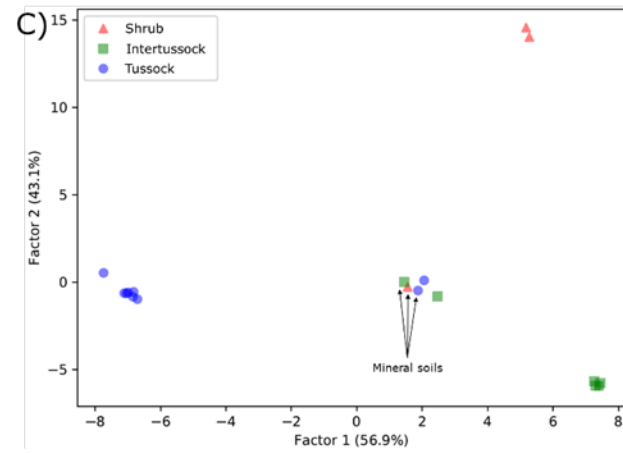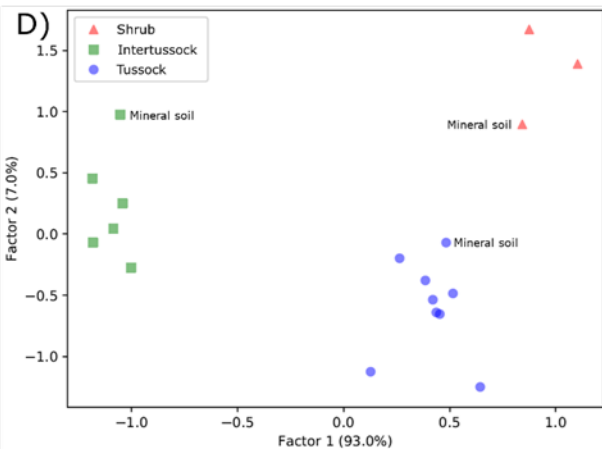

Supplement: Fig. S6 — Linear discriminant analyses of NSAF data from metaproteins defined by multiple functional annotation systems: A) GO terms, B) KEGG Orthology IDs, C) Gene Families + eggNOG descriptions, and D) Functional Groups of Gene Family + eggNOG description metaproteins. Each point is a different sample, with both organic (unlabeled points) and mineral soils (labeled) considered. The percentage is the proportion of separation due to the discriminant function. [file msystems.01238-22-s0006.pdf]

**A)**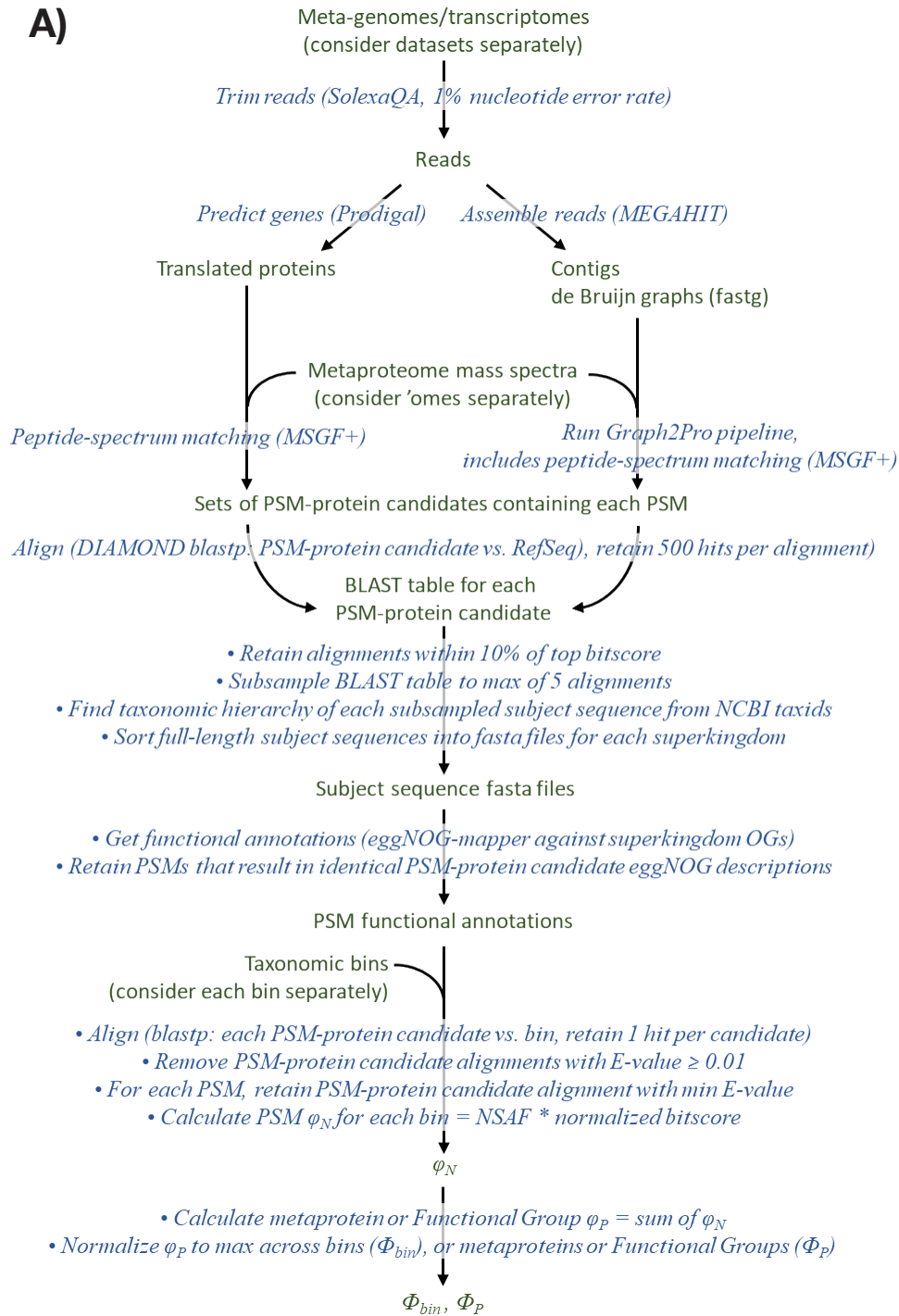**B)**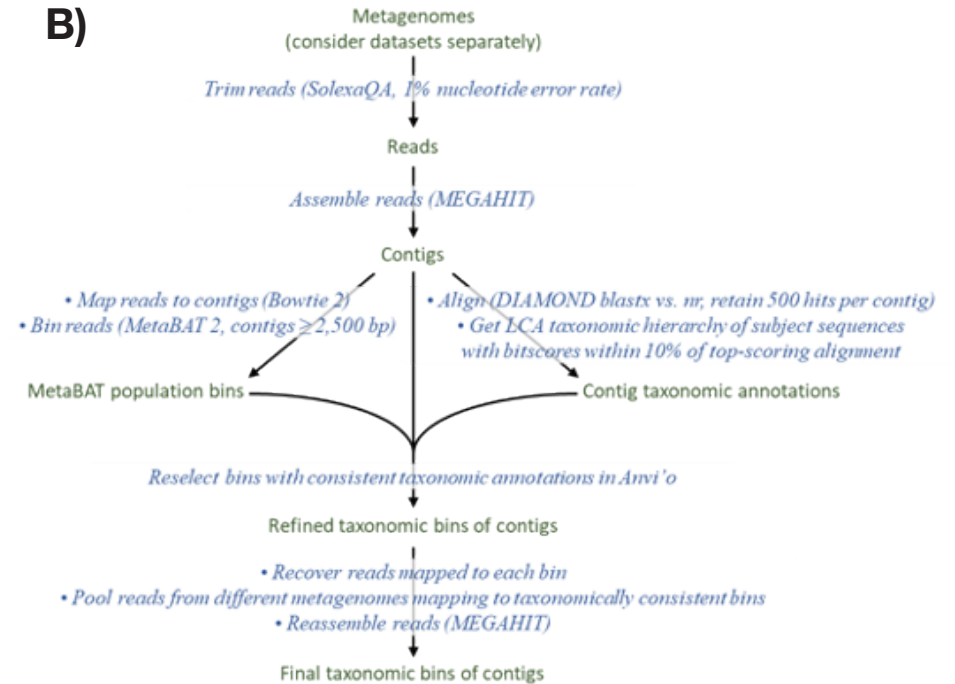

Supplement: Fig. S7 — Flowcharts of metaproteomic data analysis in ProteinExpress and of taxonomic bin construction from metagenomes. [file msystems.01238-22-s0007.pdf]
