# Supplementary material for: Metaproteomics reveals functional partitioning and vegetational variation among permafrost-affected Arctic soil bacterial communities
Source: mSystems. 2023 Jun 5;8(3):e01238-22. doi: 10.1128/msystems.01238-22 (PMC10308928; doi:10.1128/msystems.01238-22)
Supplement: Table S1 — Metaproteomic sample information. For map of sample locations, see Figure S1. “NA” in “Depth to water table (cm)” signifies that the water table was at the base of the active layer. [file msystems.01238-22-s0008.pdf]

| Sample ID | Vegetation type | Soil horizon | Northing (UTM, Zone 6 N) | Easting (UTM, Zone 6 N) | Site | Active layer depth (cm) | Depth to water table (cm) | Sampling date (in 2014) | MS/MS spectra | Peptide-spectrum matches (≤1% FDR) | Spectra with screened functional annotations | Unique screened peptide sequences | Spectra/ PSM | Spectra/ unique peptide |
|---|---|---|---|---|---|---|---|---|---|---|---|---|---|---|
| 1 | Intertussock | Organic | 7612385.993 | 406199.426 | Imnavait | 40 | 23 | Aug 11 | 38245 | 15878 | 4920 | 2099 | 7.8 | 18.2 |
| 2 | Intertussock | Organic | 7612385.993 | 406199.426 | Imnavait | 40 | 23 | Aug 11 | 39121 | 16235 | 4814 | 2223 | 8.1 | 17.6 |
| 3 | Intertussock | Organic | 7612296.508 | 405983.235 | Imnavait | 64 | 23 | Aug 11 | 36688 | 10485 | 3216 | 2081 | 11.4 | 17.6 |
| 4 | Intertussock | Organic | 7612296.508 | 405983.235 | Imnavait | 64 | 23 | Aug 11 | 36214 | 10064 | 3066 | 2050 | 11.8 | 17.7 |
| 5 | Intertussock | Organic | 7614070.44 | 393755.304 | Toolik | 61 | NA | Aug 12 | 32087 | 6052 | 1967 | 1202 | 16.3 | 26.7 |
| 6 | Intertussock | Mineral | 7614070.44 | 393755.304 | Toolik | 61 | NA | Aug 12 | 30950 | 3142 | 1079 | 372 | 28.7 | 83.2 |
| 7 | Tussock | Organic | 7612386.411 | 406199.526 | Imnavait | 53 | NA | Aug 11 | 38778 | 4898 | 1481 | 727 | 26.2 | 53.3 |
| 8 | Tussock | Organic | 7612345.005 | 406061.461 | Imnavait | 45 | NA | Aug 11 | 39321 | 15055 | 5003 | 3071 | 7.9 | 12.8 |
| 9 | Tussock | Organic | 7612345.005 | 406061.461 | Imnavait | 45 | NA | Aug 11 | 38814 | 13583 | 4341 | 2640 | 8.9 | 14.7 |
| 10 | Tussock | Organic | 7612345.005 | 406061.461 | Imnavait | 45 | NA | Aug 11 | 39415 | 13915 | 4283 | 2624 | 9.2 | 15.0 |
| 11 | Tussock | Organic | 7612345.005 | 406061.461 | Imnavait | 45 | NA | Aug 11 | 39269 | 13879 | 4207 | 2543 | 9.3 | 15.4 |
| 12 | Tussock | Organic | 7612345.005 | 406061.461 | Imnavait | 45 | NA | Aug 11 | 39583 | 12978 | 3853 | 2305 | 10.3 | 17.2 |
| 13 | Tussock | Organic | 7612345.005 | 406061.461 | Imnavait | 45 | NA | Aug 11 | 40495 | 14184 | 4417 | 2572 | 9.2 | 15.7 |
| 14 | Tussock | Organic | 7614069.165 | 393754.96 | Toolik | 60 | NA | Aug 12 | 33192 | 8621 | 2828 | 1456 | 11.7 | 22.8 |
| 15 | Tussock | Mineral | 7614069.165 | 393754.96 | Toolik | 60 | NA | Aug 12 | 33588 | 5462 | 2013 | 893 | 16.7 | 37.6 |
| 16 | Shrub | Organic | 7614490.628 | 393616.694 | Toolik | 45 | NA | Aug 10 | 33378 | 8445 | 2801 | 1670 | 11.9 | 20.0 |
| 17 | Shrub | Mineral | 7614490.628 | 393616.694 | Toolik | 45 | NA | Aug 10 | 33596 | 6674 | 1807 | 728 | 18.6 | 46.1 |
| 18 | Shrub | Organic | 7612425.334 | 406153.236 | Imnavait | 58 | 35 | Aug 11 | 36997 | 10919 | 3424 | 2292 | 10.8 | 16.1 |
