# Supplementary material for: Metaproteomics reveals functional partitioning and vegetational variation among permafrost-affected Arctic soil bacterial communities
Source: mSystems. 2023 Jun 5;8(3):e01238-22. doi: 10.1128/msystems.01238-22 (PMC10308928; doi:10.1128/msystems.01238-22)
Supplement: Table S2 — Metagenomic and metatranscriptomic sample datasets used for metaproteomic search database construction. Asterisks indicate data sets that were used in binning. [file msystems.01238-22-s0009.pdf]

**Nucleotide dataset** (ENA run accession)	**Study** (Main text reference)	**Type of sequence data**	**Location**	**Sampling date**	**Base count** (Gbp)	**Read length** (bp)
ERR1017187	80	DNA	CiPEHR, Healy	May, 2010	42.4	150
ERR1019366	80	DNA	CiPEHR, Healy	May, 2010	37.4	150
ERR1022687	80	DNA	CiPEHR, Healy	May, 2010	41.3	150
ERR1022692	80	DNA	CiPEHR, Healy	May, 2010	26.8	150
ERR1034454	80	DNA	CiPEHR, Healy	May, 2010	38.6	150
ERR1035437	80	DNA	CiPEHR, Healy	May, 2010	39.1	150
ERR1035438	80	DNA	CiPEHR, Healy	May, 2010	43.8	150
ERR1035441	80	DNA	CiPEHR, Healy	May, 2010	45.1	150
ERR1039457	80	DNA	CiPEHR, Healy	May, 2010	34.8	150
ERR1039458	80	DNA	CiPEHR, Healy	May, 2010	50.2	150
SRR5208451	55	RNA	Imnavait	June, 2013	11.9	250
SRR5208454	55	RNA	Imnavait	June, 2013	12.4	250
SRR5208455	55	RNA	Imnavait	June, 2013	29.0	250
SRR5208541	55	RNA	Imnavait	June, 2013	12.4	250
SRR5208544	55	RNA	Imnavait	June, 2013	19.1	250
SRR5208545	55	RNA	Imnavait	June, 2013	21.6	250
SRR5450431*	55	DNA	Imnavait	June, 2013	10.0	250
SRR5450432*	55	DNA	Imnavait	June, 2013	12.5	250
SRR5450434*	55	DNA	Imnavait	June, 2013	8.7	250
SRR5450438*	55	DNA	Imnavait	June, 2013	6.8	250
SRR5450631*	55	DNA	Imnavait	June, 2013	12.9	250
SRR5450755*	55	DNA	Imnavait	June, 2013	13.9	250
SRR5471030*	55	DNA	Imnavait	June, 2013	12.4	250
SRR5471031*	55	DNA	Imnavait	June, 2013	10.3	250
SRR5471032*	55	DNA	Imnavait	June, 2013	11.2	250
SRR5471221*	55	DNA	Imnavait	June, 2013	9.8	250
SRR5476649*	55	DNA	Imnavait	June, 2013	10.9	250
SRR5476651*	55	DNA	Imnavait	June, 2013	9.6	250
