# Supplementary material for: Metaproteomics reveals functional partitioning and vegetational variation among permafrost-affected Arctic soil bacterial communities
Source: mSystems. 2023 Jun 5;8(3):e01238-22. doi: 10.1128/msystems.01238-22 (PMC10308928; doi:10.1128/msystems.01238-22)
Supplement: Table S3 — Taxonomic bins constructed from metagenomic data, taxonomic ranks, and number of contigs (all ≥2,500 nt) per taxonomic bin. [file msystems.01238-22-s0010.pdf]

**Bin**	**Taxonomic rank**	**Contigs**
Rhizobiales	order	246
Pseudomonas	genus	581
Rahnella	genus	1143
Burkholderiaceae	family	501
Oxalobacteraceae	family	543
Actinobacteria	class	370
Thermoleophilia	class	130
Acidimicrobiia	class	135
Acidobacteriales	order	169
Solibacterales	order	630
Bacteroidetes	phylum	202
Myxococcales	order	89
